# Comparative Effectiveness of an In-Person and a Virtual Basic Emergency Care Instructor Course

**DOI:** 10.5334/aogh.3602

**Published:** 2022-05-20

**Authors:** Sean M. Kivlehan, Megan M. Rybarczyk, Alicia E. Genisca, Derek Lubetkin, Ramu Kharel, J. Austin Lee, Nichole Michaeli, Emilie J. Calvello Hynes, Julia Dixon, Noel Leifer, Naz Karim

**Affiliations:** 1 Department of Emergency Medicine, Brigham and Women’s Hospital, Boston, MA; 2 Department of Emergency Medicine, Perelman School of Medicine, University of Pennsylvania, Philadelphia, PA, USA; 3 Department of Emergency Medicine and Pediatrics Warren Alpert Medical School of Brown University, Providence, RI, USA; 4 Department of Emergency Medicine, Warren Alpert Medical School of Brown University, Providence, RI, USA; 5 Department of Emergency Medicine, University of Colorado School of Medicine, Aurora, CO, USA

## Abstract

**Background:**

Resource limited settings have an ongoing need for access to quality emergency care. The World Health Organization – International Committee of the Red Cross Basic Emergency Care (BEC) course is one mechanism to address this need. Training of BEC trainers has been challenging due to barriers including cost, travel logistics, scheduling, and more recently, social distancing regulations related to the coronavirus pandemic.

**Objective:**

We seek to determine if an online virtual format is an effective way to train additional trainers while overcoming these barriers.

**Methods:**

The BEC Training-of-Trainers (ToT) course was adapted to a virtual format and delivered entirely online. Participants were assessed with a multiple choice pre- and post-test and completed a course feedback form upon completion. Results from the virtual course were then compared to the results from an in-person ToT course.

**Findings:**

The in-person course pre- and post-tests were completed by 121 participants with a pre-test mean of 87% (range 60–100%) and a post-test mean of 95% (range: 75–100; p < 0.05). Virtual course pre- and post-tests by 27 participants were analyzed with a pre-test mean of 89% (range 75–100%) and a post-test mean of 96% (range: 79–100; p < 0.05). No difference in test improvements between the courses was detected (z = –0.485; p = 0.627). The course feedback was completed by 93 in-person participants and 28 virtual participants. Feedback survey responses were similar for all questions except for course length, with in-person participant responses trending towards the course being too long.

**Conclusions:**

A virtual format BEC ToT course is effective, feasible, and acceptable. When compared to an in-person course, no difference was detected in nearly all metrics for the virtual format. Utilizing this format for future courses can assist in scaling both the BEC ToT and, by extension, the BEC course globally, particularly in regions facing barriers to in-person training.

## Introduction

The unmet need for emergency care in resource limited settings has been well established [1,2]. The World Health Assembly has called for universal access to safe, high-quality, needs-based emergency care [3]. Implementation of the Basic Emergency Care (BEC) course, developed by the World Health Organization (WHO) and the International Committee for the Red Cross (ICRC) in 2018, is one mechanism to achieve this goal [4]. This course is designed to train front-line health providers in the assessment and treatment of acute and emergent conditions, particularly in resource limited settings. It has been implemented in several countries, with high-performing participants being further trained to become instructors of future classes with a Training-of-Trainers (ToT) course [5,6,7,8].

Scaling the BEC course requires significant logistic, personnel and equipment input into both the BEC and ToT courses. These challenges include the cost of holding and attending courses, the time required for attending a course, and travel logistics. Some regions of the world that could most benefit from BEC training are remote or have security challenges, which also creates a barrier to in-person training. Prior BEC implementations often limited ToT courses to small groups immediately preceding or following the BEC course itself. In an attempt to address the need for additional BEC trainers and more efficient training of trainers, a large ToT was held at the annual meeting of the American College of Emergency Physicians (ACEP) in 2019 in Denver, Colorado, with 121 participants from 22 countries. While this large ToT expanded the number of personnel trained at one time, most participants were from high income countries and per BEC delivery best practice recommendations, the new trainers would need to partner with local stakeholders at BEC implementation sites.

Furthermore, when the COVID-19 pandemic restricted travel and large gatherings globally in the spring of 2020, many prior BEC participants were forced to cancel plans to attend in-person BEC ToT courses. As a result, many BEC courses were paused or canceled. Many educational programs have successfully transitioned to a virtual format in response to COVID-19, including the ICRC Health Emergencies in Large Population (HELP) course, and a number of the London School of Hygiene and Tropical Medicine short training courses [9,10,11]. Computer based simulations have been studied and have shown to be effective in remote training of emergency care providers [12]. New training programs like Project Hope’s Virtual ToT on COVID-19 preparedness have also shown to be highly successful in knowledge gain [13].

Adjuncts to support asynchronous and remote BEC training have already been created, including a mobile application and practice cases [14,15]. In response to COVID-19 and the additional pre-existing barriers to BEC implementation, the BEC ToT course was modified to a virtual format, incorporating these existing adjuncts. A needs assessment was performed amongst current trainers who had directed both BEC and ToT courses and an adapted curriculum was created and taught virtually. Here, we report on the effectiveness, feasibility, and acceptability of a virtual BEC ToT course through a mixed methods approach as compared to a recent in-person ToT course.

## Methods

**Figure 1:**
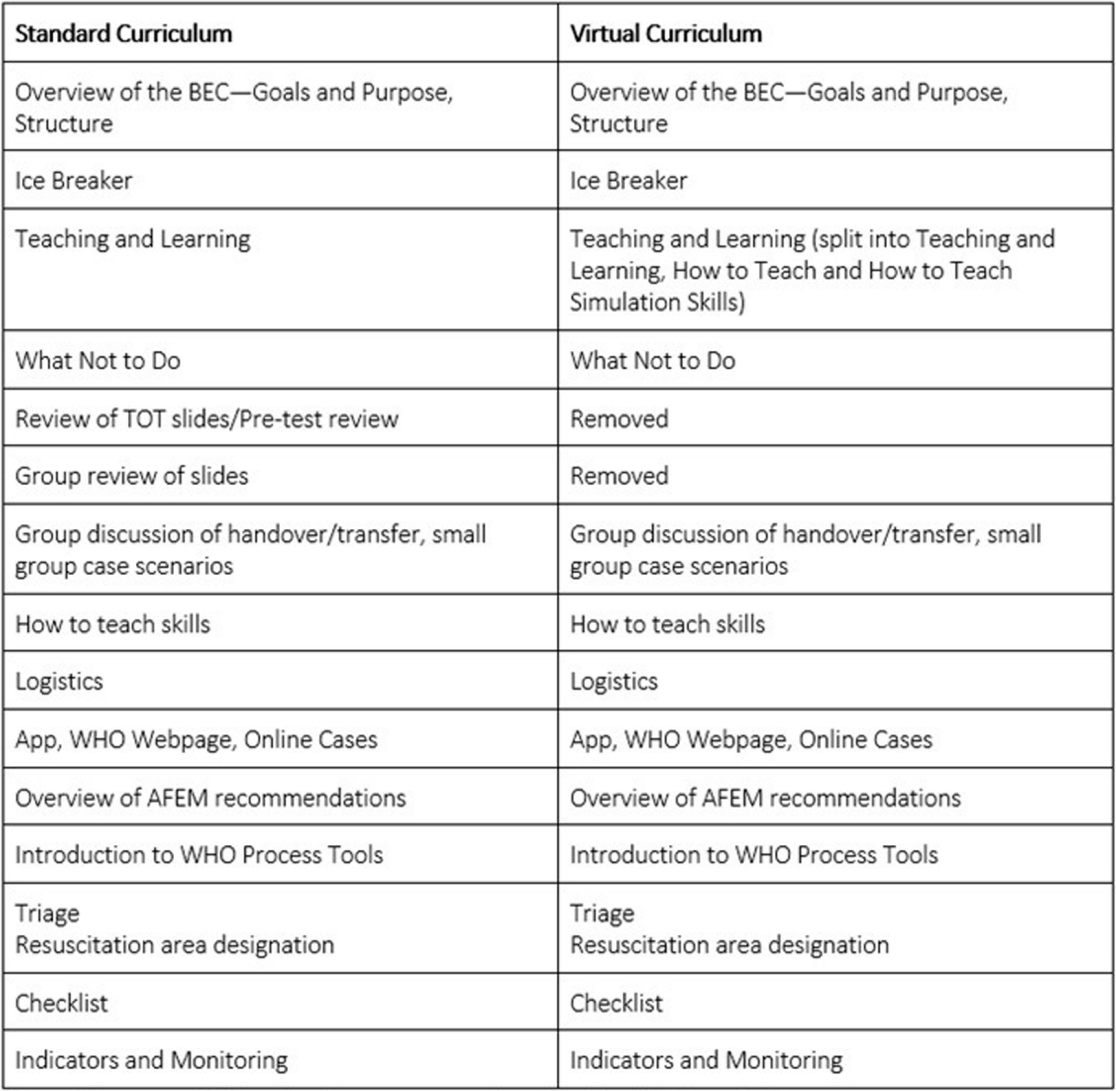
In-person ToT curriculum as compared to the virtual ToT curriculum.

**Figure 2:**
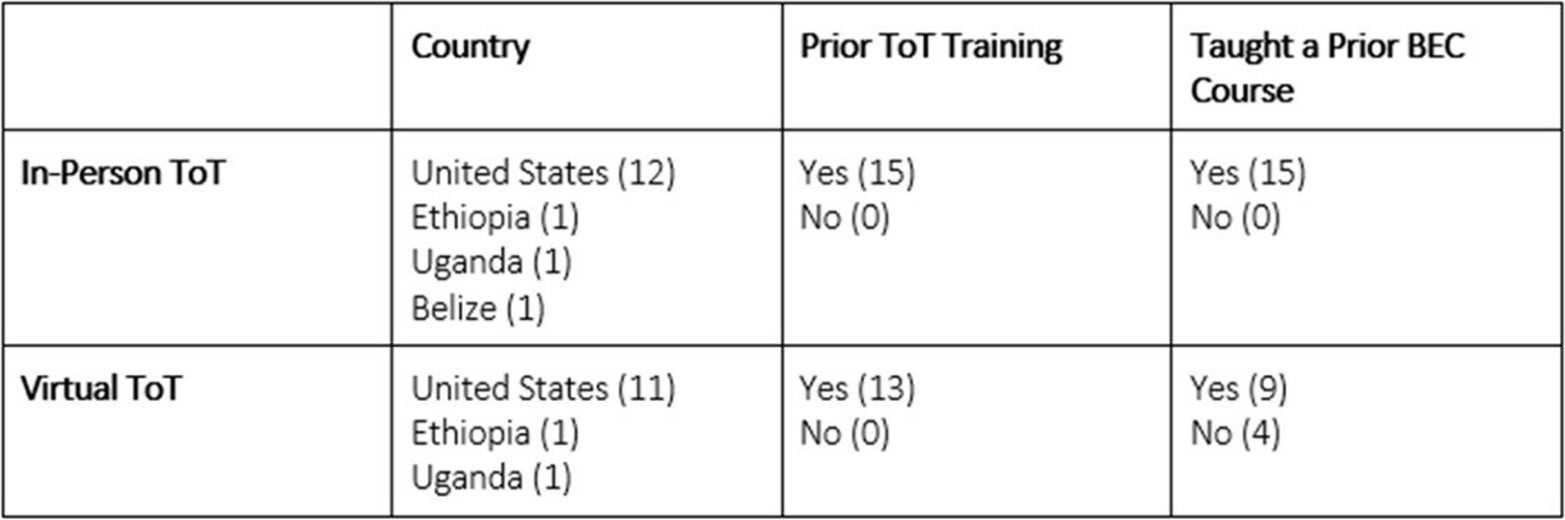
Facilitator List and competencies for both the in-person and the virtual BEC ToT course.

A needs assessment involving five BEC trainers and past participants reviewed the existing ToT curriculum and feedback from prior in-person ToT courses. Each curricular component was reviewed for inclusion in the virtual course and categorized as keep, modify, or drop. Of the 16 components, 14 were kept, none were modified, and two were dropped. The standard in-person curriculum and final virtual curriculum are shown in Figure 1. The detailed schedules from the in-person and the virtual ToT courses can be found in Appendices 1 and 2, respectively. The two-day course was taught in English, and Zoom (Zoom Video Communications, Inc., San Jose, CA) was used as the virtual platform. Moderated breakout rooms were used for small group and skill sessions. Facilitators were asked to join a WhatsApp (Facebook, Menlo Park, CA) group to allow for seamless communication throughout the day. The virtual course was taught by 13 facilitators, whose competencies are summarized in Figure 2.

De-identified participant data was prospectively collected during the virtual course and then compared to a retrospective analysis of de-identified data from an in-person ToT course that had been held in Denver, Colorado in October 2019 at the ACEP conference.

Participants for both courses were recruited via email advertisements, internet advertisements, and social media. There was no requirement for years of emergency medicine experience or prior BEC course participation. Eligible international and U.S. participants included emergency medicine physicians, residents, fellows, advanced practitioners, and nurses practicing in an acute emergent setting for at least two years. Due to the lack of uniformity of emergency medicine training outside of the United States, international participants had slightly different eligibility criteria. They were required to have had prior BEC training and to be working in the emergency setting.

Participants for both courses took the same 20 question multiple choice pre- and post-tests and completed a course feedback form upon completion of the course. Test questions were developed by the course publisher and include content on course logistics and teaching theory. In updating the content for the virtual session, content for one post-test question was not covered in the virtual course and was removed from analysis, making the virtual course post-test 19 questions. The tests and feedback forms were the standard versions developed as part of the BEC ToT training package.

The course feedback form contained seven questions structured on a 5-point Likert scale with responses ranging from 1 (least favorable) to 5 (most favorable). One question on optimal course length was structured differently with 3 being the optimal course length, and less than or more than 3 representing too short or too long of course length respectively. Participants in the virtual course had an additional three open-ended questions about opportunities for improvement, areas of success, and plans for future use of the virtual training course.

Study data were collected and managed using REDCap electronic data capture tools. Pre- and post-test scores and the course feedback form Likert scale questions were compared using a paired two-sample Student’s t-test, two-tailed (Excel version 16.0, Microsoft, Redmond, WA, and MATLAB version R2015(a), The Mathworks Inc, Natick, MA). A qualitative content analysis was performed on participant responses to the open-ended questions on the course feedback forms form each course. A content analysis was done on all responses and coded by three authors (MR, JD, SK). Inductive coding was used for each open-ended question to identify key ideas which were descriptively summarized. We used the SQUIRE checklist when writing our report [16].

Data from the in-person course was determined to be non-human subjects research by Colorado Multiple Institutional Review Board (COMIRB) protocol #21-2778. Data from the virtual course was prospectively collected and informed consent was obtained prior to participant assessment. The virtual course data plan was reviewed and approved by the Mass General Brigham IRB, protocol # 2020P003386.

## Results

### Demographics

There were 132 participants enrolled in the in-person course and 31 participants in the virtual course. Most participants in both courses were physicians, with several other health professions represented. 121 participants completed the in-person course (92.4% of enrollees), and 30 completed the virtual course (93.8% of enrollees). For the in-person course, 93 demographic and feedback forms were received (76.9% of participants), and 30 were received for the virtual course (100% of participants). Two responses from the virtual course were excluded from the analysis: one for not completing the demographics and feedback form, and one for not providing consent. The participant demographics are summarized in Table 1.

**Table 1:** Participant demographics.


	IN-PERSON COURSE (N = 121)	VIRTUAL COURSE (N = 28)

Primary Location of Current Practice	91 United States 5 Haiti 3 Pakistan 2 Colombia 2 India 2 Palestine 2 Peru 1 Australia 1 Costa Rica 1 Ecuador 1 El Salvador 1 Ghana 1 Kenya 1 Mexico 1 Panama 1 Paraguay 1 Philippines 1 Puerto Rico 1 Singapore 1 Tanzania 1 Turkey	19 United States 2 India 2 Kenya 1 Mali 1 Pakistan 1 Sri Lanka 1 Tanzania 1 United Kingdom

Professional Role	67 Physician 13 Nurse 3 Medical Student 2 Physician Assistant 1 EMT 1 Public Health Specialist 34 Unknown	26 Physician 1 Physician Assistant 1 Nurse Practitioner

Mean Years of Clinical Practice in Emergency Medicine	8.6 years (Range: 0.66–30)	9 years (Range: 0–38)

Prior Experience with a WHO BEC Course?	9% Yes/91% No	21% Yes/79% No


### Pre- and Post-Tests

The in-person course pre- and post-tests were completed by 121 participants. For the in-person course, the pre-test mean was 87% (range 60–100%) and the post-test mean was 95% (range 75–100; p < 0.05). The virtual course pre- and post-tests were completed by 30 participants, but three participants were excluded from the analysis due to lack of consent. For the virtual course, the pre-test mean was 89% (range 75–100%) and the post-test mean was 96% (range 79–100; p < 0.05). The difference between the mean pre- and post-test score improvements for the in-person and the virtual course was 2.96%. When compared with a Wilcoxon signed rank test, a difference between the pre- and post-test improvements for the in-person and virtual courses was not detected (z = –0.485; p = 0.627). (Table 2)

**Table 2:** Pre- and Post-Test mean scores.


	PRE-TEST MEAN (RANGE) N = 93	POST-TEST MEAN (RANGE) N = 27	DIFFERENCE

In-Person Course	87% (60–100%)	95% (75–100%)	p < 0.05

Virtual Course	89% (75–100%)	96% (79–100)	p < 0.05


### Course Feedback Form

The course feedback form was completed by 93 participants in the in-person course and 28 participants in the virtual course. Participants overall gave positive feedback for both the in-person and ToT courses, with the average response was greater than 4 on a scale of 0–5 with 5 being positive. There was only one question with a significant difference in responses for the in person and virtual courses and this was the course length question which used a different response scale. The average response for the course length question in both the in-person and virtual (3.7 vs 3.2 respectively p < 0.05) trend toward participants feeling that the course is too long. (Table 3)

**Table 3:** Participant feedback on the course; * optimal score 3 (<3 too short and >3 too long).


QUESTION	AVERAGE RESPONSE FOR IN-PERSON TOT (N = 93)	AVERAGE RESPONSE FOR VIRTUAL COURSE (N = 28)	DIFFERENCE

Overall, did the BEC ToT course meet your expectations?	4.6	4.3	0.3 p = 0.055

Overall, how would you rate the BEC ToT course?	n/a	4.2	n/a

Did the BEC ToT course help you to better understand the BEC curriculum and content?	4.7	4.5	0.2 p = 0.06

Do you think the BEC ToT course improved your teaching skills?	4.3	4.0	0.3 p = 0.11

How effective were the instructors as a whole?	4.6	4.4	0.2 p = 0.13

How do you feel about the BEC ToT course length?	3.7	3.2*	0.5 p < 0.05

How do you feel about the technical aspects/logistics of the virtual BEC ToT course?	4.3	4.3	0.0 p = 0.69


The first open ended question asked participants to identify unfavorable aspects of the virtual course and suggestions for improvement. Many participants stated they would recommend access to the ToT and BEC course materials and supplemental resources prior to the ToT program. A few participants noted that the organization and timing of the course could be improved, which was often related to the participants being in multiple time zones. Participants also stated they would have liked more information on the logistics of holding a BEC course and more tips for common pitfalls.

For the second open feedback question, participants described what they liked about the course. A majority of participants stated they liked the breakout sessions during the ToT course. Many added that the breakout sessions were helpful for sharing teaching experiences, receiving personal feedback, and networking. Participants also stated they enjoyed the enthusiasm, quality, and variety of course instructors. Several participants reported that they felt the virtual platform was successful.

The third question asked participants how they intended to use their training going forward. Many listed specific countries and organizations where they were going to implement BEC and use the knowledge gained in this course. Several participants specifically stated they planned to teach health care provider students in their home country.

## Discussion

The virtual format of the BEC ToT course was effective in improving knowledge as evidenced by the significant increase in test scores among participants. No difference in effectiveness was detected between the virtual ToT format and the in-person ToT format as measured by improvement in test scores and course feedback. Together, these findings suggest that a virtual ToT could be used to overcome the many barriers to in-person training and to more rapidly scale up BEC training.

Teaching the BEC ToT course virtually was feasible as evidenced by the engagement and completion rate of the course. While no major connectivity issues were encountered by participants or instructors, some individuals had to briefly restart a device or internet connection. Most curriculum components were preserved from the in-person format. The Zoom platform was useful in facilitating breakout rooms which are integrated into the software platform and allow for smaller group discussions. Breakout rooms were frequently utilized throughout the course for small group discussions, to practice teaching skills, and for skill simulations. Participants were asked to keep their video cameras turned-on throughout the entire course to ensure participant presence. Attendance was taken during each session and facilitators tracked participant presence during each session to verify participants met the requirements for course completion. The WhatsApp groups allowed for real time coordination and problem solving between facilitators and for clear and accurate directions to participants.

Responses in the course feedback forms indicate that the virtual ToT format was acceptable to participants. No significant difference was detected in responses related to the quality of the course except for with regards to course length. While both in-person and virtual course participants felt the course was the optimal length, in-person participant responses significantly trended to a feeling that the course was too long. Both courses were held over two days; the in-person ToT course was 12.5 hours long and the virtual course was 13.5 hours long. However, the in-person course was split to have only 2.5 hours on the first day and 10 hours on the second day while the virtual course was a more even split of 7 and 6.5 hours, which may account for this difference in responses. Participants felt that early access to course materials would have improved the experience. They also commented on the opening of the virtual course, which is likely related to the initial difficulties some had joining the online session. While these were resolved quickly and did not appear to affect the overall delivery of the course, future virtual courses should emphasize the initial logon experience. The breakout rooms and facilitator variety were seen as strengths, which supports the need for a low student to facilitator ratio and the importance of small group sessions.

There are pros and cons to both the in-person and the virtual formats. Positives for the in-person course include interactive teaching and learning while being in-person, ability to perform skills fully hands-on with standardized equipment, and the networking benefits of down time. These are balanced by the negatives of an in-person course, such as the financial costs of attendance in travel and conference fees and the need to schedule additional clinical time off.

The financial cost of travel, housing, and meals for in-person trainings can easily exceed $500 to $1500 for domestic and international conferences, respectively [17,18]. These costs do not include conference and training fees and the financial burden of time away from work. While many academic institutions provide their employees with reimbursement for conference attendance, in-person conferences and trainings inevitably exclude participants who cannot afford these costs. In-person courses may disproportionately exclude potential participants from low- and middle-income countries, whom the BEC ToT seeks to engage [19].

A virtual course allows for lower costs as participants and facilitators do not need to travel, nor are classrooms or catering required. Scheduling is more flexible as participants can take less time off than would be required for domestic or international travel. Additionally, the course does not need to be attached to a large conference as was previously done to facilitate attendance. While the in-person course discussed here was particularly diverse, this was largely due to one-time scholarship funding for many of the international participants, and it is unlikely other in-person courses could maintain such diversity without ongoing funding. Virtual training can allow participants from regions where travel is logistically difficult, such as areas in a humanitarian crisis or with ongoing conflict, to attend.

In addition, virtual courses limit the impact of travel on the environment, and, in the time of the coronavirus pandemic, concerns of disease transmission. Virtual courses allow for safe continued training during the coronavirus pandemic, which can translate to future public health events that may require social distancing. By providing a virtual training format, this course can overcome many existing barriers, increase opportunities for the targeted providers to attend, and expedite scaling of the course globally.

There are downsides to virtual training that we identified, including time zone challenges for a live course delivered to people around the world and internet connectivity issues. The virtual ToT was delivered to participants in eight countries on four continents. Future virtual courses can mitigate some of the time zone differences through recorded sessions of selected content. Participants are required to obtain their own equipment to practice skills in a virtual course which places an additional burden on them and can make corrective action by facilitators more challenging. Networking, which is a benefit to attending courses such as this, can be more challenging when virtual but improved through the use of breakout room activities and ice-breaking sessions.

### Limitations

There are limitations to this analysis of the virtual format of the BEC ToT course. This BEC ToT was advertised in English on listservs used by global health providers and taught entirely in English, which may have excluded non-English speaking participants. The language of instruction is an important consideration, since the course aims to reach a diverse global audience. However, by involving international providers, this course may indirectly benefit non-English speaking providers who participate in future BEC courses. Future research should include a needs assessment of providers with diverse language backgrounds, and additional virtual courses could be offered in other languages. The course was largely taught by North American based facilitators, and the lack of an asynchronous option may have led to lower participation from providers in significantly different time zones. Additionally, participants were required to have access to high-speed internet and electronic devices. While the participants did not have any major issues with connectivity, these requirements may represent barriers to engaging participants in settings without stable electricity or internet connections. In-person courses may still be necessary to reach remote populations. However, trainers who have completed the virtual BEC ToT may provide a valuable link to providers in more remote and resource limited settings.

## Conclusion

The findings here suggest that the virtual ToT course is at least as effective as the in-person format with the added benefit of overcoming barriers to training. Despite the limitations and challenges of conducting a virtual training, the overall success of the course supports plans for future virtual courses. Virtual training can rapidly increase the number of qualified BEC course instructors which will exponentially increase the number of health care providers who can be reached by future BEC courses. This will hopefully translate into improved access to care and better patient outcomes around the world. Future efforts should be aimed at evaluating higher-level educational outcomes from virtual training and to determine if the BEC course can also be delivered in a virtual or hybrid format.

## Additional Files

The additional files for this article can be found as follows:

10.5334/aogh.3602.s1 Appendix 1. Agenda and Schedule for In-Person Basic Emergency Care Training-of-Trainers Course.

10.5334/aogh.3602.s2 Appendix 2. Agenda and Schedule for Virtual Basic Emergency Care Training-of-Trainers Course.

10.5334/aogh.3602.s3 SQUIRE Checklist. Completed Revised Standards for Quality Improvement Reporting Excellence (SQUIRE 2.0) Checklist.
