# Supplementary material for: Comparative Effectiveness of an In-Person and a Virtual Basic Emergency Care Instructor Course
Source: Ann Glob Health. 2022 May 20;88(1):35. doi: 10.5334/aogh.3602 (PMC9122011; doi:10.5334/aogh.3602)
Supplement: Appendix 1. — Agenda and Schedule for In-Person Basic Emergency Care Training-of-Trainers Course. [file agh-88-1-3602-s1.pdf]

WHO/ICRC Basic Emergency Care Facilitator Course

PARTICIPANT AGENDA FOR THE DAY
October 25th and 26th, 2019

Friday, October 25, 2019

4:30 – 5:00	Registration
5:00 – 5:15	Welcome and Introductions - Dr. Emilie Calvello Hynes
5:15 – 5:30	Goals and Purpose of BEC - Dr. Andi Tenner
5:30 – 5:45	Ice breaker
5:45 – 6:15	Structure of the BEC and set up of the course - Dr. Shama Patel
6:15 – 6:35	Teaching and learning - Dr. Julia Dixon
6:35 – 6:50	What not to do - Dr. Sean Kilvehan
6:50– 7:00	Review the day tomorrow and wrap up - Dr. Emilie Calvello Hynes

Saturday, October 26, 2019 Schedule

7:00 - 7:30	Registration
7:30 – 7:45	Welcome by ACEP Representative
7:45 – 9:00	Review and practice of ABCDE slides (mid size group activity)
9:00 – 10:15	Review and practice of Trauma slides (small group activity)
10:15 – 10:30	*Break*
10:30 – 11:30	Shock, DIB, AMS lectures (small group activity)
11:30 – 12:15	Lunch
12:15 - 12:30	Handover/Transfer - Dr. Shama Patel
12:30 – 12:45	Small Group Case Scenarios - Dr. Andi Tenner
12:45– 1:15	Practice Small Group Case Scenarios (small group activity)
1:30 – 2:00	Teaching Skills - Dr. Sean Kilvehan
2:00 – 2:15	*Break*
2:15 – 3:15	Skills teaching practice (small group activity)
3:15– 3:45	Feedback on skills teaching (mid size group activity)
3:45 – 4:15	MCQs and documentation of the course - Dr. Andi Tenner
4:15 – 4:45	Post Test and Evaluations
4:45 - 5:00	Close
