# Supplementary material for: Comparative Effectiveness of an In-Person and a Virtual Basic Emergency Care Instructor Course
Source: Ann Glob Health. 2022 May 20;88(1):35. doi: 10.5334/aogh.3602 (PMC9122011; doi:10.5334/aogh.3602)
Supplement: Appendix 2. — Agenda and Schedule for Virtual Basic Emergency Care Training-of-Trainers Course. [file agh-88-1-3602-s2.pdf]

SAEM/GEMA WHO BEC TOT Schedule

DAY 1 — Wednesday November 18, 2020
9:00 – 16:00 EST

Time	Session	Facilitator
9:00-9:30	Overview of the BEC: Goals, Purpose, Structure	Dr. Naz Karim
9:30-9:45	Icebreaker (breakout)	Dr. Stephen Dunlop (introduction)
9:45-10:30	Teaching and Learning	Dr. Elizabeth DeVos
10:30-10:45	What Not To Do	Dr. Alicia Genisca
10:45-11:00	Break	
11:00-11:45	BEC Tools	Dr. Branden Skarpiak
11:45-12:45	How to Give a Lecture (breakout/practice)	Dr. Alicia Genisca (introduction)
12:45-13:30	Lunch Break	
13:30-14:00	How to Teach Skills	Dr. Austin Lee
14:00-15:00	Simulation/Skills Practice (breakout)	Dr. Derek Lubetkin (introduction)
15:00-15:15	Logistics	Dr. Shama Patel
15:15-15:30	Break	
15:30-15:45	Overview of IFEM Recommendations	Dr. Alicia Genisca
15:45-16:00	Wrap Up and Close	Dr. Naz Karim

DAY 2 — Thursday November 19, 2020
8:30 – 15:00 EST

Time	Session	Facilitator
8:30-9:00	App, WHO Webpage, Online Cases	Dr. Ramu Kharel
9:00-9:30	Triage	Dr. Branden Skarpiak
9:30-10:30	Handover/Transfer (group discussion, small group case scenarios)	Dr. Stephen Dunlop (lecture)
10:30-10:45	Break	
10:45-11:15	Introduction to WHO Process Tools	Dr. Elizabeth DeVos
11:15-11:45	Checklist	Dr. Ramu Kharel
11:45-12:15	Resuscitation and Designation	Dr. Sean Kivlehan
12:15-13:00	Lunch Break	
13:00-13:30	Indicators and Monitoring	Dr. Sean Kivlehan
13:30-14:30	Simulation/Skills Practice (breakout)	Dr. Derek Lubetkin (introduction)
14:30-14:45	Wrap Up and Close	Dr. Naz Karim
14:45-15:00	Evaluations/Post Test	

Task to Complete: https://bit.ly/3nhdw1d

(Please complete Post-Test immediately at end of course)
